# Cumulative Inflammatory Load Is Associated with Short Leukocyte Telomere Length in the Health, Aging and Body Composition Study

**DOI:** 10.1371/journal.pone.0019687

**Published:** 2011-05-13

**Authors:** Aoife O'Donovan, Matthew S. Pantell, Eli Puterman, Firdaus S. Dhabhar, Elizabeth H. Blackburn, Kristine Yaffe, Richard M. Cawthon, Patricia L. Opresko, Wen-Chi Hsueh, Suzanne Satterfield, Anne B. Newman, Hilsa N. Ayonayon, Susan M. Rubin, Tamara B. Harris, Elissa S. Epel, for the Health Aging and Body Composition Study

**Affiliations:** 1 Department of Psychiatry, University of California San Francisco, San Francisco, California, United States of America; 2 San Francisco Veterans Affairs Medical Center, San Francisco, California, United States of America; 3 University of California, Berkeley-University of California, San Francisco Joint Medical Program, Berkeley and San Francisco, California, United States of America; 4 Department of Psychiatry and Behavioral Sciences, Stanford University, Stanford, California, United States of America; 5 Department of Biochemistry and Biophysics, University of California San Francisco, San Francisco, California, United States of America; 6 Department of Neurology, University of California San Francisco, San Francisco, California, United States of America; 7 Department of Epidemiology and Biostatistics, University of California San Francisco, San Francisco, California, United States of America; 8 Department of Human Genetics, University of Utah, Salt Lake City, Utah, United States of America; 9 Department of Environmental and Occupational Health, University of Pittsburgh, Pittsburgh, Pennsylvania, United States of America; 10 Department of Medicine, University of California San Francisco, San Francisco, California, United States of America; 11 Department of Preventive Medicine, University of Tennessee, Memphis, Tennessee, United States of America; 12 Department of Epidemiology, University of Pittsburgh, Pittsburgh, Pennsylvania, United States of America; 13 Laboratory for Epidemiology, Demography and Biometry, National Institute on Aging, Bethesda, Maryland, United States of America; Massachusetts General Hospital, United States of America

## Abstract

**Background:**

Leukocyte telomere length (LTL) is an emerging marker of biological age. Chronic inflammatory activity is commonly proposed as a promoter of biological aging in general, and of leukocyte telomere shortening in particular. In addition, senescent cells with critically short telomeres produce pro-inflammatory factors. However, in spite of the proposed causal links between inflammatory activity and LTL, there is little clinical evidence in support of their covariation and interaction.

**Methodology/Principal Findings:**

To address this issue, we examined if individuals with high levels of the systemic inflammatory markers interleukin-6 (IL-6), tumor necrosis factor-α (TNF-α) and C-reactive protein (CRP) had increased odds for short LTL. Our sample included 1,962 high-functioning adults who participated in the Health, Aging and Body Composition Study (age range: 70–79 years). Logistic regression analyses indicated that individuals with high levels of either IL-6 or TNF-α had significantly higher odds for short LTL. Furthermore, individuals with high levels of both IL-6 and TNF-α had significantly higher odds for short LTL compared with those who had neither high (OR = 0.52, CI = 0.37–0.72), only IL-6 high (OR = 0.57, CI = 0.39–0.83) or only TNF-α high (OR = 0.67, CI = 0.46–0.99), adjusting for a wide variety of established risk factors and potential confounds. In contrast, CRP was not associated with LTL.

**Conclusions/Significance:**

Results suggest that cumulative inflammatory load, as indexed by the combination of high levels of IL-6 and TNF-α, is associated with increased odds for short LTL. In contrast, high levels of CRP were not accompanied by short LTL in this cohort of older adults. These data provide the first large-scale demonstration of links between inflammatory markers and LTL in an older population.

## Introduction

Telomeres, the DNA-protein complexes that cap the ends of chromosomes and protect against genomic instability, contain variable-length tracts of telomeric DNA. Leukocyte telomere length (LTL) is increasingly recognized as an index of biological age that predicts incidence of age-related diseases [1], [2], as well as all-cause and disease-specific mortality in diverse cohorts of older adults [1], [3], [4], [5], [6]. In fact, older adults with below average LTL have more than threefold increased risk for early mortality [4], [6]. Long LTL is also a predictor of years of healthy living, an outcome that integrates self-perceived functional status and duration of survival [7]. Importantly, telomere shortening is not just an index, but also a potential mechanism underlying some aspects of biological aging because short telomere length can lead to cessation of mitosis with consequent loss of ability for cell replenishment and, in some cellular settings, genomic instability, end-to-end chromosome fusion, and apoptosis as well as harmful DNA damage signaling and mitochondrial dysfunction [8], [9], [10], [11]. Notwithstanding the utility of LTL in predicting mortality and longevity, there is little clinical evidence regarding modifiable factors associated with and potentially driving leukocyte telomere shortening.

Inflammatory activity is frequently proposed as a contributor to biological aging in general, and leukocyte telomere shortening in particular [5], [12], [13], [14]. Accumulating evidence suggests a causal role for inflammation in the pathogenesis of multiple age-related diseases including cancer, atherosclerosis, autoimmune disorders, diabetes, and neurodegenerative diseases [15], [16], [17], [18], [19], [20], [21]. Elevated inflammatory activity could accelerate leukocyte telomere shortening by promoting cell turnover and replicative senescence [22], and by inducing the release of reactive oxygen species that damage telomeric DNA via oxidative stress [23]. One complication is that inflammatory cytokines including tumor necrosis factor-α (TNF-α) can both inhibit and promote activity of telomerase [24], [25], [26], the cellular enzyme primarily responsible for lengthening telomeres [27]. On the other hand, *in vitro* research on various cell types, but not including normal leukocytes, indicates that an accumulation of senescent cells with critically short LTL may produce pro-inflammatory factors [28], [29], hich could in turn contribute to an increased inflammatory load.

Observations of short LTL in small samples of patients with inflammatory diseases including hepatitis C, liver cirrhosis, chronic kidney disease and chronic obstructive pulmonary disease provide preliminary support for the proposal that inflammation accelerates leukocyte telomere shortening [5], [14], [30], [31]. Specifically, in these studies, patients with inflammatory disorders exhibited shorter LTL cross-sectionally compared with healthy individuals [31], [32], and shorter telomeres in cells proximal to disease-related inflammatory activity [14], [30]. Additionally, elevations in disease-specific and systemic inflammatory markers have been associated with short LTL in patients [5]. Although there are plausible pathways by which inflammation could accelerate telomere shortening even in adults without chronic disease, little is known about *in vivo* relationships between markers of inflammation and LTL in healthy older adults.

In the present study, we examined if elevated inflammatory activity, indexed by high levels of the systemic inflammatory cytokines interleukin-6 (IL-6) and TNF-α and the acute phase protein C-reactive protein (CRP), is associated with increased risk for short LTL in the ‘Health, Aging and Body Composition’ (Health ABC) cohort, a large sample of well-functioning men and women aged 70–79 years. Our sample included 1,962 Health ABC participants who had complete data available for our primary analyses. We hypothesized that participants with high levels of IL-6 and/or TNF-α and/or CRP would be more likely than participants with lower levels of these inflammatory markers to have short LTL, and that the combination of high IL-6, high TNF-α and high CRP, as an index of a high cumulative inflammatory load, would confer the greatest odds for short LTL.

## Results

In spite of the restricted age range of the sample, age was significantly associated with shorter LTL (*r* = −.07, *p* = .002) and higher levels of TNF-α (*r* = .07, *p* = .003). However, age was negatively associated with levels of CRP (*r* = −.07, *p* = .001) and was not associated with levels of IL-6 (*r* = .03, *p* = .19). Inflammatory markers were significantly and positively associated with one another. However, while the effect size for the relationship between IL-6 and CRP was medium to large (*r* = .47, *p*<.001), relationships between IL-6 and TNF-α (*r* = .27, *p*<.001) as well as between TNF-α and CRP (*r* = .11, *p*<.001) had smaller effect sizes. Table 1 summarizes the baseline characteristics of the study population stratified by LTL tertile. As predicted, participants in the bottom tertile for LTL had the highest level of IL-6 and TNF-α, but there were no differences between groups in levels of CRP.

**Table 1:** Sample characteristics by leukocyte telomere length (LTL) tertile.

		LTL Tertile			
		Short	Middle	Long	*p value*
LTL (base pairs)		≤4260	4261–5280	>5280	
Age (years), M (SD)		74.0 (2.8)	73.5 (2.9)	73.4 (2.9)	<.01**
Race (Black), n (%)		239 (36.5)	274 (41.8)	271 (41.6)	.09
Sex (female), n (%)		255 (39.0)	330 (50.3)	398 (61.0)	<.01**
Site (Memphis), n (%)		324 (49.5)	367 (56.0)	293 (44.9)	<.01**
Body mass index (kg/m^2^)		27.6 (4.8)	27.4 (4.9)	27.3 (4.7)	.59
Smoking status, n (%)					
	Current	72 (11.0)	73 (11.1)	66 (10.1)	
	Former	319 (48.8)	305 (46.5)	270 (41.4)	
	Never	263 (40.2)	278 (42.4)	316 (48.5)	.04*
Sleep (hours/night)		6.9 (1.3)	6.9 (1.4)	6.8 (1.3)	.79
Alcohol (drinks/week), n (%)					
	0	303 (46.3)	333 (50.7)	328 (50.3)	
	<1	142 (21.7)	144 (22.0)	129 (19.8)	
	1–7	160 (24.5)	135 (20.6)	142 (21.8)	
	>7	49 (7.5)	44 (6.7)	53 (8.1)	.46
Exercise (kcal/kg/week)		85.9 (73.5)	85.7 (73.1)	82.6 (64.0)	.93
Chronic condition, n (%)		444 (67.9)	448 (68.2)	464 (71.2)	.38
Recent infection, n (%)		43 (6.6)	46 (7.0)	52 (8.0)	.61
Anti-inflammatory medications, n (%)		344 (52.6)	356 (54.3)	332 (50.9)	.48
Statins, n (%)		89 (13.6)	79 (12.0)	97 (14.9)	.32
Income, n (%)					
	<10 k	68 (10.4)	86 (13.1)	88 (13.5)	
	10 k–25 k	262 (40.0)	272 (41.5)	242 (37.1)	
	25 k–50 k	217 (33.2)	205 (31.3)	218 (33.4)	
	>50 k	107 (16.4)	93 (14.2)	104 (16.0)	.38
Education, n (%)					
	<HS	156 (23.9)	166 (25.3)	123 (18.9)	
	HS	191 (29.2)	184 (28.1)	186 (28.4)	
	>HS	307 (46.9)	306 (46.7)	343 (52.6)	.05*
Interleukin-6: Mdn (IQR)		1.9 (1.3–3.0)	1.8 (1.3–2.8)	1.7 (1.2–4.5)	.05*
Tumor necrosis factor-α: Mdn (IQR)		3.4 (2.7–4.4)	3.2 (2.5–4.1)	3.0 (2.3–3.8)	<.01**
C-reactive protein: Mdn (IQR)		1.6 (1.0–3.0)	1.6 (0.9–3.0)	1.74 (1.0–3.3)	.17

Notes. HS = high school; IQR = interquartile range; LTL = leukocyte telomere length; M = mean; Mdn = Median; n = number of participants; SD = standard deviation; p-values for comparisons of means were calculated using one-way ANCOVA tests for continuous variables, and chi-squared tests for categorical variables. One-way ANCOVAs were calculated using log-transformed values because variances were not equal between groups. However, reported means and medians are based on raw data.

### Inflammatory Activity and LTL

In our primary analytic model, we found that the odds of having short LTL (i.e., LTL in the bottom tertile or ≤4260 bp) were significantly higher for those participants who had high (i.e., top tertile) levels of either IL-6 (≥2.39 pg/mL), TNF-α (≥3.72 pg/mL) or CRP (≥2.51 mg/L). In these analyses, the results of which are summarized in Table 2, groups were not mutually exclusive, such that the same individual could be represented in multiple groups if he or she had high levels of IL-6, TNF-α and CRP. Adjusting for a wide range of potential confounds and covariates, the odds for short LTL were significantly higher in those who had either IL-6 levels in the top tertile (OR = 1.3, 95% CI = 1.1–1.7) or TNF-α levels in the top tertile (OR = 1.5, 95% CI = 1.2–1.9). The highest odds for short LTL were observed in those participants who had high levels of both IL-6 and TNF-α (OR = 1.8, CI = 1.3–2.4). In contrast, the addition of high levels of CRP did not confer increased odds for short LTL, as indexed by the lack of association between CRP and LTL (OR = 1.1, 95% CI = 08–1.4), and by the roughly equal odds of having short LTL for those who had high levels of CRP in addition to high levels of IL-6 and TNF-α (OR = 1.7, CI = 1.1–2.6).

**Table 2:** Systemic inflammatory markers as predictors of short leukocyte telomere length.

	Unadjusted		Adjusted for age, gender and race		Adjusted for all covariates	
	OR	(95% CI)	OR	(95% CI)	OR	(95% CI)
IL-6	1.3	(1.0–1.6)*	1.3	(1.0–1.6)*	1.3	(1.0–1.7)*
TNF-α	1.6	(1.3–2.1)**	1.5	(1.2–1.91)**	1.5	(1.2–1.9)**
CRP	0.9	(0.7–1.1)	1.1	(0.86–1.4)	1.1	(0.8–1.4)
IL-6+TNF-α	1.9	(1.4–2.6)**	1.7	(1.3–2.4)**	1.8	(1.3–2.4)**
IL-6+CRP	1.1	(0.9–1.5)	1.3	(0.9–1.7)	1.3	(0.9–1.7)
TNF-α+CRP	1.6	(1.2–2.2)**	1.7	(1.2–2.4)**	1.7	(1.2–2.4)**
IL-6+TNF-α+CRP	1.7	(1.2–2.5)**	1.7	(1.1–2.5)**	1.7	(1.1–2.6)**

Notes.

*indicates *p*<.05 and

**indicates *p*<.01;

OR refers to odds ratio based on logistic regression; 95% CI is the 95% confidence interval; IL-6 = Interleukin-6; TNF-α = Tumor necrosis factor-α; CRP = C-reactive protein.

We additionally ran the same models using the established high-risk clinical cutoff (>3 mg/L) for CRP [33]. While individuals who had CRP above this clinical cutoff had marginally higher odds for short LTL, this was not significant in any of the models, including when we only adjusted for telomere batch (OR = 1.3, CI = 1.0–1.7). Thus, individuals with CRP in either the top tertile in the sample or above the recognized clinical cutoff did not appear to have significantly increased odds for short LTL.

To further examine the observed associations of IL-6 and TNF-α with short LTL and to compare mutually exclusive groups instead of potentially overlapping groups, we excluded CRP from our model and compared the odds for short LTL among four mutually exclusive groups of participants: those with both high IL-6 and high TNF-α; only high IL-6; only high TNF-α; and neither high IL-6 nor high TNF-α. These analyses were conducted while statistically controlling for established risk factors and potential confounds and with both high IL-6 and high TNF-α as our reference group. Results indicated that the combination of both high IL-6 and high TNF-α levels was associated with roughly double the odds for short LTL compared with having neither high IL-6 nor high TNF-α (OR = 0.52, CI = 0.37–0.72). Furthermore, the combination of high IL-6 and high TNF-α was associated with significantly higher odds for short LTL compared with having only IL-6 high (OR = 0.57, CI = 0.39–0.83) or only TNF-α high (OR = 0.67, CI = 0.46–0.99). Finally, mean LTL adjusted for site, age, gender, and race was 201 base pairs shorter in participants with high levels of both IL-6 and TNF-α (*M* = 4701.93 bp, *SE* = 68.07) compared with that of participants who did not have high levels of both of these (*M* = 4902.21 bp, *SE* = 28.20), and this difference in absolute LTL was significant, *F*(1,1937) = 7.35, *p* = .007.

## Discussion

Short LTL, a potential index of biological age, is associated with increased risk for age-related diseases as well as early all-cause and disease-specific mortality [3], [4], [5], [6]. The present research study is to date the largest to demonstrate an association between elevated inflammatory activity and short LTL. Moreover, the present study is the first to indicate that the combination of high IL-6 and TNF-α is associated with increased odds for short LTL, over and above the odds associated with having high levels of only one of these markers. Of note, having high levels of both IL-6 and TNF-α conferred almost twice the odds of being in the bottom tertile for LTL compared with having lower levels of both markers. In contrast, having high levels of CRP, as indexed by CRP levels either in the top tertile for the sample or above the established clinical cutoff, was not associated with increased odds for short LTL. Importantly, all of our results held when controlling for the contribution of numerous established risk factors for short LTL and potential confounds. Together with the observed causal relationships between inflammatory activity and LTL in animal and *in vitro* studies, these data provide preliminary evidence that adjunct anti-inflammatory therapies could potentially prevent accelerated leukocyte telomere shortening or protect against the negative effects of short LTL in older adults.

We included IL-6, TNF-α and CRP as three separate measures of systemic inflammatory activity in our study. Although all of these inflammatory markers were significantly correlated with one another, relationships between them were not strong (*r*'s≤.47). IL-6 and CRP were the most strongly associated of the inflammatory markers, sharing 22% of variance. Given that IL-6 is a major inducer of CRP production by hepatocytes [34], [35], the larger effect size for the relationship between these markers is not unexpected. However, high levels of IL-6, but not high levels of CRP, were associated with increased odds of short LTL in the sample. There is evidence that IL-6 is necessary but not sufficient to induce CRP synthesis [36], and also evidence that human coronary artery smooth muscle cells may produce CRP and that IL-6 is not a necessary inducer of CRP synthesis by these cells [37]. Thus, although IL-6 is an inducer of CRP and their concentrations are correlated, the physiological actions of IL-6 and CRP are not expected to be identical. The present findings, which need to be further investigated, suggest that in the case of LTL, these two factors do not have the same effects. Notably with regard to our overall findings, IL-6 and TNF-α shared only 7% of variance, supporting the formulation that circulating levels of these cytokines reflect different aspects of the inflammatory response. Thus, while each of these cytokines may contribute to leukocyte telomere shortening by promoting cell turnover and replicative senescence, inducing oxidative stress, and regulating telomerase activity [22], [23], [24], [25], [26], chronic exposure to the cumulative load of both high IL-6 and high TNF-α may have the greatest impact on the rate of leukocyte telomere shortening.

Equally plausible, however, is the emerging hypothesis that participants with short LTL in our study had the greatest proportion of accumulated senescent bodily cells overall, including fibroblasts and epithelial cells, which could plausibly contribute to the observed higher levels of IL-6 and TNF-α in this group [28], [38]. Given that TNF-α and IL-6 may inhibit programmed cell death in specific cell types [39], [40], high levels of these cytokines could even contribute to the maintenance of senescent cells in the system and hence the continued production of pro-inflammatory factors. However, the cross-sectional design of our study precludes drawing conclusions about the causal direction in the relationship between inflammatory activity and LTL.

In previous research, the cumulative load of high IL-6, high TNF-α and high CRP was found to confer the greatest risk for cardiovascular events in the Health ABC cohort [7]. However, CRP did not likewise contribute to the predictive value of the inflammatory markers with regard to LTL. Our finding of no association between CRP and LTL is in line with a previous finding of no associations between CRP and LTL in post-menopausal women, and in ‘Cardiovascular Health Study’ participants who were older than 73 years [7]. However, this finding does not support our hypothesis that higher levels of all measures of inflammatory activity would be associated with shorter LTL. Previous research has demonstrated that although CRP is reliably associated with cardiovascular disease (CVD) outcomes, CRP does not appear to be causally involved in the development of CVD [41]. Furthermore, CRP is less predictive of CVD in older compared with younger adults [42], [43] and CRP levels were not predictive of cardiovascular events in a sample of high-risk Japanese adults in whom IL-6 was an independent predictor of such events [44]. However, it is possible that CRP would be associated with LTL in older populations with other diseases, such as inflammatory diseases characterized by very high levels of CRP. Our findings in relation to CRP may have particular relevance in clinical settings where CRP is the most commonly used index of inflammatory activity because they suggest that pro-inflammatory cytokine concentrations may serve as complementary indices of inflammation.

The present findings must be interpreted in the context of several limitations. First, the cross-sectional design of the present study precludes causal interpretations. Second, the narrow age range of our sample of older adults (70–79 years) is both a strength and weakness of our study. While this narrow age range allows us to maximize power to detect associations between inflammatory activity and LTL without many of the confounding factors associated with a sample of wider age range (e.g., cohort effects, hormonal effects), the findings of the present study may not be generalizable to other stages of the lifespan. Moreover, anti-inflammatory medications were common in our sample of older adults. Given the wide range of medications that have anti-inflammatory effects and the variability in dosage across individuals, our statistical control for anti-inflammatory medications is likely to be insufficient but is the best option currently available. There has been debate about the relative merits of different methods used to assay LTL and the Q-PCR method has been subject to criticism [45], [46]. However, strong associations between LTL as assessed by the Southern blot method and Q-PCR method have been documented [47], and the coefficient of variation of the LTL assay for this study was low at 4%. In addition, some findings related to LTL in Health ABC have been replicated in a cohort who had LTL measured with the traditional Southern blot technique [48]. Finally, it should be noted that effect sizes were small in this study and caution is therefore advised in the interpretation of the results pending future studies in independent cohorts.

Data from this large cohort of older adults indicates links between inflammatory activity and biological aging. In particular, the present data indicate that the cumulative load of high IL-6 and high TNF-α is accompanied by increased risk for short LTL. The present data also replicate a previous finding of no association between CRP and LTL in older adults, and suggest that systemic inflammatory cytokines may have more relevance to leukocyte telomere shortening than the more commonly used inflammatory marker CRP. In sum, older adults with high levels of inflammatory activity may be at increased risk for accelerated leukocyte telomere shortening, and those with short LTL may have increased risk for diseases with an inflammatory etiology.

## Methods

### Study Population

Study participants for this investigation included 1,962 well-functioning men and women aged 70–79 years who participated in Health ABC. Participants were identified from a random sample of white Medicare beneficiaries and all age-eligible black community residents in designated ZIP code areas surrounding Pittsburgh and Memphis. To be eligible for participation in Health ABC, participants had to report no difficulty in walking one-quarter mile (0.5 km) or climbing 10 stairs without resting. Exclusion criteria included reported difficulty performing basic activities of daily living, obvious cognitive impairment, inability to communicate with the interviewer, intention of moving within 3 years, or participation in a trial involving a lifestyle intervention. Additionally, of the 3.075 participants who took part in Health ABC, our sample of 1,962 includes only participants who had complete data on LTL, inflammatory markers and all covariates included in our analysis. Our sample was not significantly different from excluded participants with missing data on age or site, but they were less likely to be female (50.1% of subsample versus 54% of non-sample, *p* = .04) and less likely to be Black (40% of subsample versus 44.7% of non-sample, *p* = .01). All participants gave written informed consent. The Institutional Review Boards at the University of Pittsburgh, the University of Tennessee and the University of California, San Francisco approved the protocol. Baseline data were collected from 1997 to 1998.

### LTL Measurement

Quantitative polymerase chain reaction (Q-PCR) was used to measure LTL in the genomic DNA of peripheral leukocytes by determining the ratio of telomere repeat copy number to single-copy gene copy number (T/S ratio) in study samples relative to a reference sample [47], [49]. The Q-PCR assay was conducted on three separate DNA samples per participant, and average LTL was calculated as the mean value of these triplicates. Each T/S value was later converted to number of base pairs (bp) by multiplying the T/S value by the known LTL of the reference DNA, which is a pooled sample of DNAs from several normal Utah whites aged 65 years and older. The slope of the linear regression line through a plot of T/S ratio (the x axis) versus mean TRF length (the y axis) is the number of base pairs of telomeric DNA corresponding to a single T/S unit. All samples were measured in triplicate and the mean value of the triplicates was used in analyses. The coefficient of variation for this assay is 4% and results obtained with the Q-PCR method are strongly associated with the traditional terminal restriction fragment length index of LTL obtained by Southern blot technique [47]. DNA was available for 2,880 of the 3,075 individuals who participated in Health ABC, and LTL was successfully measured in 2,721 cases.

### Inflammatory Markers

Blood samples for inflammatory markers were obtained in the morning (median time was 9:19 AM; interquartile range was from 8:49 AM to 9:52 AM). IL-6 and CRP levels were measured in serum and TNF-α levels were measured in plasma. Tubes for IL-6 and CRP were serum separator tubes containing no anticoagulant. Tubes for TNF-α were citrated tubes containing 0.5 mL of 3.8% sodium citrate. After processing, the specimens were aliquoted into cryovials, frozen at −70°C, and shipped to the Core Laboratory at the University of Vermont. Serum IL-6 levels and plasma TNF-α levels were measured in duplicate by enzyme-linked immunosorbent assay (ELISA) kits from R&D Systems (Minneapolis, MN). The detectable limit for IL-6 (by HS600 Quantikine kit) was .10 pg/mL, and for TNF-α (by HSTA50 kit) was .18 pg/mL. Plasma levels of CRP were also measured in duplicate by ELISA based on purified protein and polyclonal anti-CRP antibodies (Calbiochem, San Diego, CA). The CRP assay was standardized according to the World Health Organization First International Reference Standard, with a sensitivity of 0.08 µg/mL. Measures of IL-6, TNF-α and CRP were missing for 162, 203, and 38 participants respectively. Intra and inter-assay coefficients of variation for inflammatory markers were respectively: 10% and 15% for IL-6; 16% and 15% for TNF-α; and 8% and 5% for CRP.

### Covariates

Covariates were selected a priori based on previous research and included characteristics associated with inflammatory markers and LTL in previous research. Such a priori selection of covariates has been validated for logistic regression procedures [50], [51]. The telomere batch number corresponds to a particular run of the Q-PCR assay used to measure LTL. Because there is some variability between assays, we controlled for telomere batch in all LTL analyses, constructing a dummy variable for each batch to enter into regression models. Sociodemographic covariates included study site, age, gender, and race, as well as education (less than high school; high school; more than high school) and income (<$10 k; $10–25 k; $25–50 k; >$50 k). BMI was calculated as weight in kilograms divided by height in meters squared. Behavioral factors including smoking status (current, former, never), average alcohol use during the past year (0, <1, 1–7 or >7 drinks/week), exercise (total kilocalories burned per week) and hours of sleep per night on average were assessed in the baseline interview. Participants were asked whether they had experienced symptoms of respiratory infections within the last 2 weeks. The baseline presence of chronic illnesses including lung disease, heart disease (including myocardial infarction, angina pectoris, and congestive heart failure), stroke, diabetes mellitus, broken hip, and arthritis was adjudicated using standardized algorithms considering various sources of information: self-report of a medical diagnosis, medication use, and results of screening tests from a clinical examination where appropriate. All medications regularly taken in the past 2 weeks were recorded and coded according to the Iowa Drug Information System (IDIS) code [52]. Using this drug inventory, the daily use of antiinflammatory drugs (IDIS codes 2808 or 5208) and statins (IDIS code 2406) was assessed.

Our primary models were run with three different sets of covariates. First, adjusting only for telomere batch; second, adjusting for telomere batch, as well as potential confounds including study site, age, gender and race; and third, adjusting for telomere batch, as well as potential confounds and other potential covariates including study site, age, gender, race, income, education, BMI, smoking status, alcohol use, exercise, sleep, chronic disease, recent respiratory illness, and the use of anti-inflammatory drugs or statins.

### Statistical Analysis

Logistic regression and analysis of covariance were used to test our primary hypothesis that high levels of inflammatory activity would be associated with increased odds for short LTL, as indexed by the odds of having LTL in the bottom tertile in the sample and by LTL in base pairs, respectively. For the purpose of these analyses, participants with values in the upper tertile of the sample were classified as “high” for inflammatory markers and those with values in the lower tertile of the sample were classified as “short” for LTL. We also examined if having CRP levels above the clinical cutoff of >3 mg/L was associated with increased odds for short LTL.

Logistic regression analyses were used to examine if participants with high levels of IL-6, TNF-α and CRP alone or in combination would be more likely than participants with lower levels of these inflammatory markers to have short LTL. In our first set of analyses, groups were not mutually exclusive, such that the same individual could be represented in multiple groups if they had high levels of IL-6, TNF-α or CRP. Based on the results of these analyses, we excluded CRP from our model and conducted follow-up analyses to examine if the odds for short LTL in participants with high levels of both IL-6 and TNF-α was significantly higher than that of participants with high levels of only IL-6, high levels of only TNF-α, or high levels of neither IL-6 nor TNF-α. Thus, this second group of logistic regression analyses were conducted on mutually exclusive groups. All models were run with the three different sets of covariates as described in the covariates section. Analysis of covariance was used to compare mean differences in base pairs of telomeres between those with and without high levels of inflammatory markers.

In order to compare characteristics of the sample by telomere length tertile we performed analysis of variance and Kruskal-Wallis rank tests for continuous variables, and chi-squared tests for categorical variables. In order to analyze differences in LTL and cytokines by gender, linear regressions were used. Due to unacceptable levels of skewness, the variables IL-6, TNF-α and CRP were log-transformed when used in correlations and linear regressions. All analyses were performed using Stata version 9.2 and SPSS version 18.0.
